# Successful Management of Airway and Esophageal Foreign Body Obstruction in a Child

**DOI:** 10.1155/2019/6858171

**Published:** 2019-12-24

**Authors:** Naoki Yogo, Chiaki Toida, Takashi Muguruma, Masayasu Gakumazawa, Mafumi Shinohara, Ichiro Takeuchi

**Affiliations:** Department of Emergency Medicine, Yokohama City University Graduate School of Medicine, Yokohama, Japan

## Abstract

Foreign body asphyxia is a serious clinical problem with high morbidity and mortality rates. It is relatively common among children, especially those younger than 3 years, because they have a high risk of aspirating foreign bodies owing to their tendency to place objects in their mouth and lack of a well-developed swallowing reflex. Moreover, the neurologic outcome after out-of-hospital cardiac arrests (OHCA) in pediatric patients remains generally poor. Here, we report an unusual pediatric case of asphyxial OHCA caused by foreign bodies obstructing the airway, complicating esophageal foreign body, with a neurologically favorable outcome. This case highlights the importance of adequate treatment for pediatric patients with OHCA, as well as the prompt and efficient management for pediatric patients with foreign bodies obstructing the airway and esophagus.

## 1. Introduction

Asphyxia is the leading cause of out-of-hospital cardiac arrests (OHCA) due to external causes in Japan [1]. Although the incidence rate of asphyxia as an external cause is lower than that of internal causes (71.5%) [2], foreign body asphyxia is a serious clinical problem with high morbidity and mortality rates. It is relatively common among children, especially those younger than 3 years, because they have a high risk of aspirating foreign bodies owing to their tendency to place objects in their mouth and lack of a well-developed swallowing reflex [3–6]. Moreover, the neurological outcome after OHCA in pediatric patients remains generally poor [7–10]. Here, we report an unusual pediatric case of asphyxial OHCA caused by foreign bodies obstructing the airway, complicating esophageal foreign body, with a neurologically favorable outcome. This case report might be useful for fellow physicians to recognize the need to prepare a system of management, including medical staff education, to improve the outcomes of pediatric OHCA patients with both airway and esophageal foreign body obstruction.

## 2. Case Presentation

A 1-year-11-month old boy with no medical history was found to be struggling to breathe and unable to speak by his mother at home. She suspected that the patient's airway was obstructed by a foreign body and performed the Heimlich maneuver. Because the foreign body could not be removed and the patient began losing consciousness, she promptly called the emergency medical services (EMS). The EMS telecommunicator did not recognize the OHCA, and so the operator did not instruct the patient's mother to initiate bystander cardiopulmonary resuscitation (CPR); as a result, bystander CPR was not performed. Six minutes after placing the emergency call, the patient was found to have pulseless electrical activity and the EMS personnel initiated CPR. During the resuscitation, bag ventilation and chest compressions were performed. The patient was resuscitated after 20 min of cardiac arrest before arrival at our hospital.

**Figure 1:**
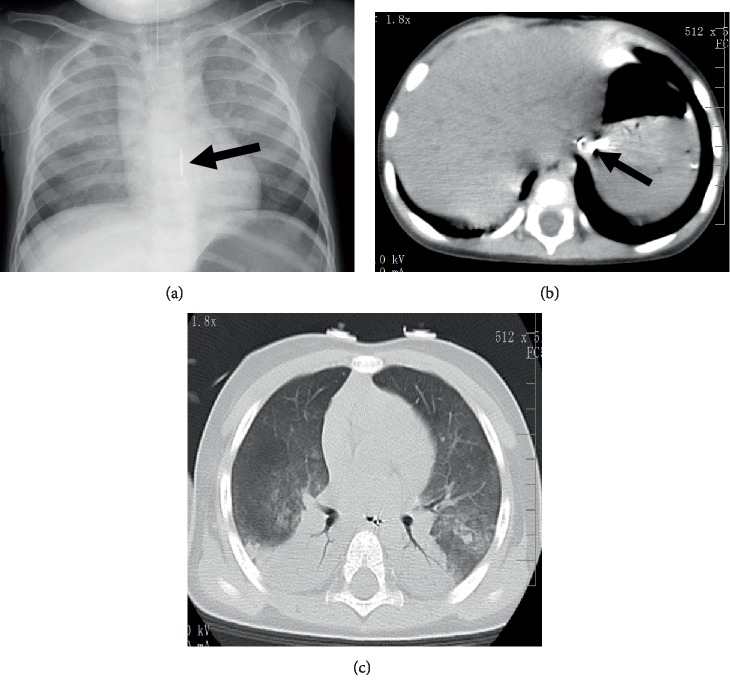
Imaging findings at hospital admission. (a) Chest X-ray; (b, c) chest computed tomography.

Upon arrival at the emergency department, coma (Glasgow Coma Scale score 3/15 [E1V1M1]) and a small amount of intraoral bleeding were noted. The respiratory rate was 36 breaths/min and oxygenation was slightly impaired with a peripheral capillary oxygen saturation level of 94% on room air. The blood pressure was 129/71 mmHg, and the heart rate was 159 beats/min. Arterial blood gas analysis revealed respiratory and metabolic acidosis and the rise in lactic acid value (pH 6.89, P_a_CO_2_ 58.3 mmHg, P_a_O_2_ 69.3 mmHg, base excess −22.7 mEq/L, HCO_3_^−^ 10.8 mEq/L, and lactate 14.4 mmol/L). Upon laboratory examination, the following results were above normal: white blood cell count (25,730/*μ*L) and D-dimer (23.1 *μ*g/mL) levels. A chest X-ray and computed tomography scan showed a foreign body at the lower esophagus and bilateral pulmonary consolidation (Figure 1).

**Figure 2:**
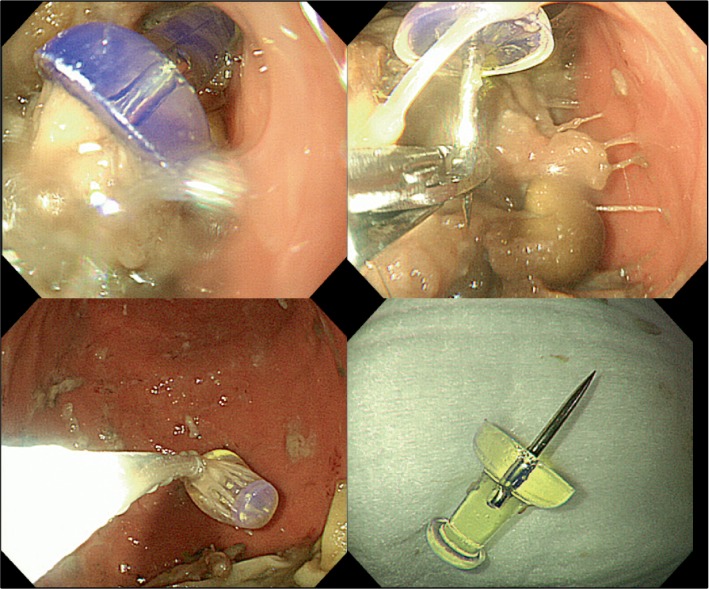
Upper gastrointestinal endoscopy findings on foreign body extraction.

Because the patient presented with coma and aspiration pneumonia, tracheal intubation was performed. At that time of intubation, the foreign body was not detected in the oral and upper airways. Therapeutic hypothermia with a target temperature of 34°C was begun after one and a half hours of arrival at our hospital, for the critical management of post-cardiac arrest syndrome. After stabilizing his general condition, the patient underwent upper gastrointestinal endoscopy, which revealed a piece of pushpin in the stomach (Figure 2). A pair of basket forceps was used to remove the foreign body in order not to injure the mucous membrane of the esophagus with the sharp tip of the pushpin. Follow-up endoscopy performed just after the removal of the pushpin revealed no mucosal injury.

After a hypothermic period of 36 h and rewarming period of 24 h, on post-OHCA day 4, the patient was weaned from the ventilator and tracheal extubation was performed. On post-OHCA day 6, the patient was transferred from the intensive care unit to the general ward. On post-OHCA day 11, the patient was discharged from the hospital to his home with pediatric cerebral performance category 1, defined as a condition without subsequent neurological complications.

## 3. Discussion

We have reported a pediatric case of OHCA due to foreign bodies obstructing the airway, complicated by an esophageal foreign body, and with no subsequent neurological complications. This case highlights the importance of adequate treatment of pediatric patients with OHCA, as well as the prompt and efficient management of pediatric patients with foreign bodies obstructing the airway and esophagus.

Previous studies have shown that children younger than 3 years are at high risk for airway and esophageal foreign bodies [3–6]. The mortality rate of pediatric patients with foreign body aspiration ranges from 1.8% to 3.4% [5, 11]. Therefore, foreign body aspiration is a common and life-threatening problem in pediatrics, resulting in a high mortality rate and severe neurological consequences. The most commonly affected part of the airway is the right bronchial tree and the laryngeal obstruction which occurred in our case was rare (3%) [12]. In laryngeal obstruction, a sharp-edged or irregular-form tool could easily obstruct and lead to life-threatening problems such as cardiac arrest [13]. On clinical examination, although diminished breath sounds on one side of the chest and/or stridor was the common finding, an episode of sudden choking was the most popular presentation among younger children [3]. Moreover, children presented with a slightly shorter interval between asphyxia and cardiac arrest. Therefore, on identifying airway obstruction, the Japan Resuscitation Council recommends (1) an emergency call to the EMS, (2) the Heimlich maneuver, and (3) CPR if the patient has lost consciousness. The 2015 national guidelines of the Japan Resuscitation Council emphasized the importance of early identification of OHCA and oral instruction of bystander CPR by EMS telecommunicators. In this case, because the EMS telecommunicator did not recognize OHCA and did not instruct bystander CPR, the patients could not undergo CPR until EMS arrival after 6 min. It is said that the survival rate of patients who did not recognize OHCA by an EMS telecommunicator in an emergency call was lower than that of patients who recognized OHCA [14]. It is important to optimize the ability of EMS telecommunicators and increase the recognition rate of OHCA in order to further improve the outcome of patients after OHCA. There remain other issues regarding the prevention of OHCA caused by foreign body airway obstruction, such as the “chain of survival”. Preventive measures to protect children from foreign body aspiration and to prevent mortality and morbidity have been reported as parental and caregiver education and modification of the design of products that could cause foreign body aspiration [6]. Reporting cases of foreign body aspiration, such as this one, might be useful for the prevention of future cases.

In order to prevent death and complications due to foreign body aspiration, it is also essential to diagnose and remove the foreign body promptly. However, previous studies reported on the risk during bronchoscopy or endoscopy among pediatric patients [6]. This means that success with foreign body extraction depends on the expertise of the bronchoscopist, endoscopist, and intensivist with considerable experience in this delicate procedure among pediatric patients [3]. In this case, it seemed that the esophageal foreign body happened during CPR for OHCA due to airway obstruction resulting from asphyxia. Moreover, this patient swallowed the sharp-edged tool and was clinically unstable due to aspiration pneumonia and post-cardiac arrest syndrome. As a result, high-quality management was needed, including foreign body removal and the management of respiratory and circulatory states during the procedure.

Therefore, emergency centers that see a lot of critically-ill patients such as ours, should develop a system of management, including medical staff education, for patients with both airway and esophageal foreign body obstruction. Moreover, our patient required post-resuscitation therapy including hypothermia treatment.

## References

[1] Kitamura T., Kiyohara K., Sakai T. (2014). Epidemiology and outcome of adult out -of hospital cardiac arrest of noncardiac origin in Osaka: a population-based study. *British Medical Journal Open*.

[2] Kitamura T., Iwami T., Kawamura T. (2012). Nationwide improvements in survival from out-of-hospital cardiac arrest in Japan. *Circulation*.

[3] Casalini A. G., Majori M., Anghinolfi M. (2013). Foreign body aspiration in adults and in children: advantages and consequences of a dedicated protocol in our 30-year experience. *Journal of Bronchology & Interventional Pulmonology*.

[4] Shlizerman L., Mazzawi S., Rakover Y., Ashkenazi D. (2010). Foreign body aspiration in children: the effects of delayed diagnosis. *American Journal of Otolaryngology*.

[5] Shah R. K., Patel A., Lander L., Choi S. S. (2010). Management of foreign bodies obstructing the airway in children. *Archives of Otolaryngology–Head & Neck Surgery*.

[6] Brkic F., Umihanic S., Altumbabic H. (2018). Death as a consequence of foreign body aspiration in children. *Medical Archives*.

[7] Goto Y., Funada A., Nakatsu-Goto Y. (2015). Neurological outcomes in children dead on hospital arrival. *Critical Care*.

[8] Goto Y., Funada A., Goto Y. (2016). Duration of prehospital cardiopulmonary resuscitation and favorable neurological outcomes for pediatric out-of-hospital cardiac arrests: a nationwide, population-based cohort study. *Circulation*.

[9] Michiels E., Quan L., Dumas F., Rea T. (2016). Long-term neurologic outcomes following paediatric out-of-hospital cardiac arrest. *Resuscitation*.

[10] Michelson K. A., Hudgins J. D., Monuteaux M. C., Bachur R. G., Finkelstein J. A. (2018). Cardiac arrest survival in pediatric and general emergency departments. *Pediatrics*.

[11] Zhijun C., Fugao Z., Niankai Z., Jingjing C. (2008). Therapeutic experience from 1428 patients with pediatric tracheobronchial foreign body. *Journal of Pediatric Surgery*.

[12] Eren S., Balci A. E., Dikici B., Doblan M., Eren M. N. (2003). Foreign body aspiration in children: experience of 1160 cases. *Annals of Tropical Paediatrics*.

[13] Lima J. A. (1989). Laryngeal foreign bodies in children: a persistent, life-threatening problem. *The Laryngoscope*.

[14] Berdowski J., Beekhuis F., Zwinderman A. H., Tijssen J. G., Koster R. W. (2009). Importance of the first link: description and recognition of an out-of-hospital cardiac arrest in an emergency call. *Circulation*.

